# Size profile of cell-free DNA: A beacon guiding the practice and innovation of clinical testing

**DOI:** 10.7150/thno.42565

**Published:** 2020-03-26

**Authors:** Jiping Shi, Runling Zhang, Jinming Li, Rui Zhang

**Affiliations:** 1 National Center for Clinical Laboratories, Beijing Hospital, National Center of Gerontology; Institute of Geriatric Medicine, Chinese Academy of Medical Sciences, Beijing, People's Republic of China; 2 Graduate School, Peking Union Medical College, Chinese Academy of Medical Sciences, Beijing 100730, People's Republic of China; 3 Peking University Fifth School of Clinical Medicine, Beijing Hospital, Beijing, People's Republic of China; 4 Beijing Engineering Research Center of Laboratory Medicine, Beijing, People's Republic of China

**Keywords:** cell-free DNA, size profile, mechanism, clinical application, quality control

## Abstract

Cell-free DNA (cfDNA) has pioneered the development of noninvasive prenatal testing and liquid biopsy, its emerging applications include organ transplantation, autoimmune diseases, and many other disorders; size profile of cfDNA is a crucial biological property and is essential for its clinical applications. Therefore, a thorough mastery of the characteristic and potential applications of cfDNA size profile is needed.

**Methods**: Based on the recent researches, we summarized the size profile of cfDNA in pregnant women, tumor patients, transplant recipients and systemic lupus erythematosus (SLE) patients to explore the common features. We also concluded the applications of size profile in pre-analytical phases, analytical phases for novel assays, and preparation of quality control materials (QCMs).

**Results**: The size profile of cfDNA shared common features in different populations, and was distributed as a “ladder” pattern with a dominant peak at ~166 bp. However, cfDNA entailed slightly discrepant characteristics due to specific tissues of origin. The dominant peaks of fetal and maternal cfDNA fragments in pregnant women were at 143 bp and 166 bp, respectively. The plasma cfDNA in tumor patients, transplant recipients, and SLE patients had a peak of around 166 bp. In pre-analytical phases, size profile served as a vital indicator to judge the eligibility of specimens, thus ensuring the successful implementation of assays. More importantly, the size profile had the potential to enrich short fragments, calculate fetal fraction, detect fetal abnormalities, predict tumor progress in analytical phase and to guide the preparation of QCMs.

**Conclusions**: Our finding summarized the characteristics and potential applications of cfDNA size profile, providing clinical researchers with novel assays by the extensive application of cfDNA.

## Introduction

Cell-free DNA (cfDNA) was first discovered in human serum and subsequently extracted from urine, cerebrospinal fluid, and pleural fluid in the past few decades 1, 2. The cfDNA derived from fetal and tumor tissues has greatly facilitated the development of noninvasive prenatal testing (NIPT), liquid biopsy, and other potential applications, thus holding promise for noninvasive detection of fetal abnormalities or tumor characterization at an early stage. Recently, cfDNA has been shown to be non-randomly fragmented and to have a specific pattern of nucleosome distribution with associated preferred end signatures 3, 4; therefore, a comprehensive understanding of the features, mechanisms, and potential use of the size profile of cfDNA is a promising scientific area.

**Figure 1:**
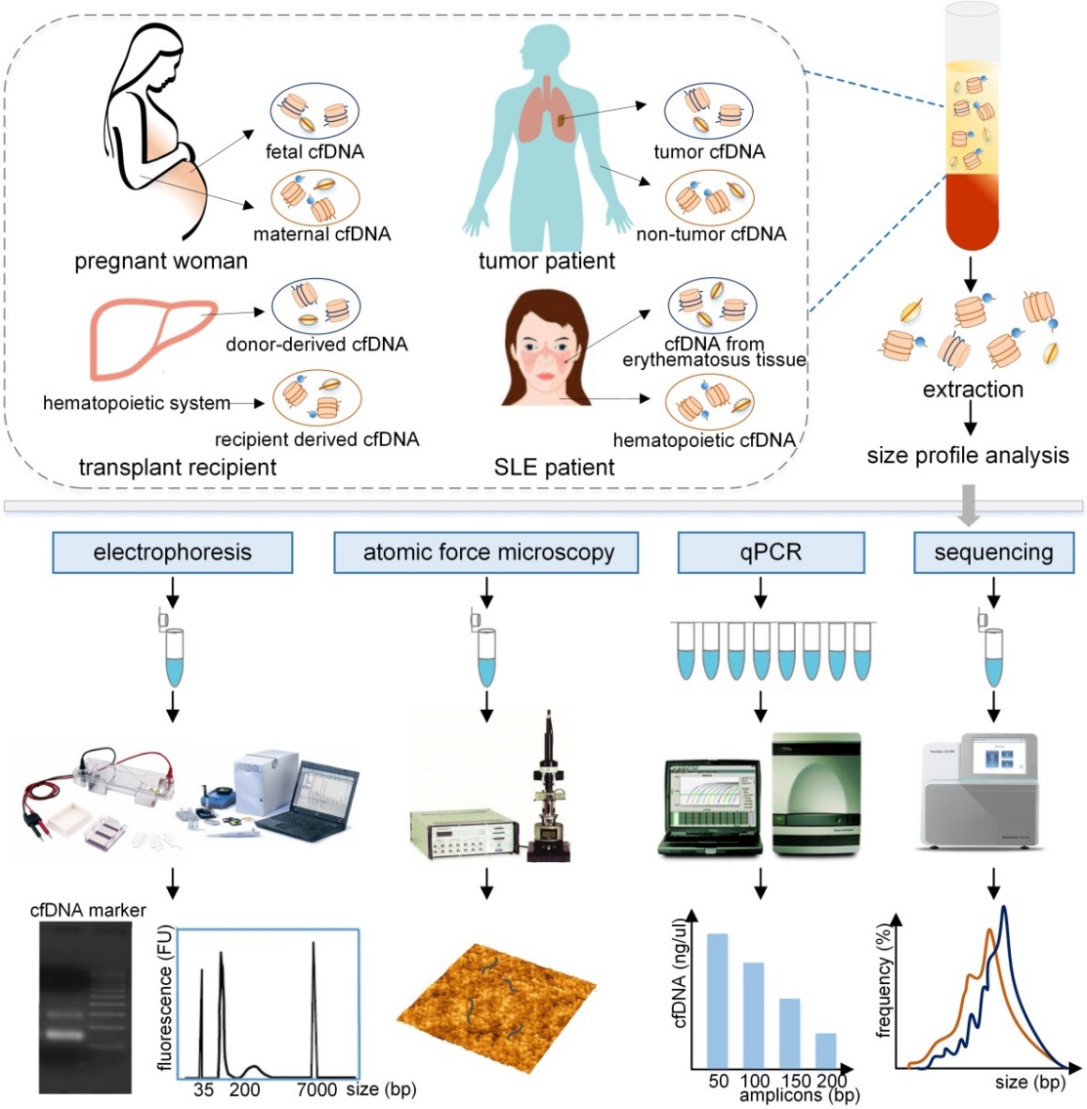
** Evaluation of the size profile of cfDNA in different populations by various analytical approaches.** The plasma cfDNA in pregnant women contains fetal and maternal cfDNA, primarily derived from fetal tissues and maternal hematopoietic system. Similarly, tumor cfDNA and non-tumor cfDNA originated from the plasma cfDNA in tumor patients. Donor-derived cfDNA and recipient derived cfDNA, and cfDNA from lupus erythematosus tissue and hematopoietic cfDNA were obtained from transplant recipients, and SLE patients, respectively. These cfDNA fragments, which are typically bound with histones or transcription factors, are released into the peripheral blood. After extraction, the size profile of cfDNA fragments can be assessed by using electrophoresis, atomic force microscopy, qPCR with different amplicons, and sequencing, producing different forms of results to represent the size profile of cfDNA. qPCR: quantitative real-time PCR; SLE: systemic lupus erythematosus.

As a crucial biological property of cfDNA, size profile has been assessed by a variety of methods, such as gel electrophoresis, atomic force microscopy (AFM), quantitative real-time PCR (qPCR), and massively parallel sequencing (MPS), the last two are relatively robust and precise methods (Figure 1) 5. Another approach to evaluate size profile is the DNA integrity, which is conducted by using qPCR with long and short amplicons (e.g., >300 bp and <100 bp) 6, and is calculated as the ratio of the number of long to short DNA fragments. Various studies have demonstrated that fetal cfDNA is shorter than maternal cfDNA in pregnant women and, therefore, fetal cfDNA can be used to detect fetal abnormalities. The precise assessment of size profile in cancer patients was slightly different based upon various methods, types and stages of tumors, and positions of cfDNA 7, 8 and should be carefully discussed. In addition, the size profile of cfDNA has been widely applied in several fields, which include quality control in laboratory practice, enrichment of the short DNA fragments, the detection of the fetal fraction (FF) and fetal abnormalities in NIPT, the prediction of tumor progression in liquid biopsies, and allograft damage in transplantation. Hence, a summary of the cfDNA size profile in different populations and its applications are required to demonstrate the significance of cfDNA.

In this review, we first discuss and summarize the size profile and mechanism of cfDNA in different populations. We then focus on the applications of size profile in pre-analytical and analytical phases in the laboratory and guidance in the preparation of quality control materials (QCMs). We believe that a thorough understanding of size profile and its relevant implementations in cfDNA could assure reliable results in clinical practice and provide valuable information for the extensive development of assays.

## Size profile of cfDNA in different populations

### Pregnant women

The analysis of size profile of fetal cfDNA in maternal plasma is simple and straightforward because fetal and maternal cfDNA fragments are easy to distinguish by using specific approaches: (1) The SRY gene, which is located only on chromosome Y, can be applied to analyze fetal cfDNA in the plasma of a pregnant woman with a male fetus 9. (2) The methylation status of specific genes is a distinguishable marker. For example, the CpG sites of the SERPINB5 gene promoter from placental tissues are hypomethylated, but almost completely methylated in the maternal blood cells; the former is the source of fetal cfDNA while the latter releases maternal cfDNA 10.

With the recent developments in detection technologies, analysis of the size profile of cfDNA fragments is becoming more precise (Table 1). Li *et al.* first found that fetal cfDNA was shorter than maternal cfDNA; the former was usually <300 bp while the latter was >1000 bp by using fluorescent PCR 11. The authors concluded that fetal cfDNA could be enriched by size selection with the length threshold of ~300 bp 11, which was significant for the development of NIPT. However, subsequent studies have proven that the precise length of fetal and maternal cfNDA in the study of Li *et al.* was not accurate. Chan *et al.* reported that the plasma cfDNA in pregnant women mainly ranged between 145-201 bp by qPCR using different amplicons 9. By employing paired-end sequencing, cfDNA in pregnant women had a dominant peak at around 162 bp and a minor peak at around 340 bp 12. Fetal cfDNA identified by chromosome Y sequences was rarely longer than 250 bp but was mostly present in sizes of <150 bp 12. Another sequencing study also reported similar results 13, confirming the feasibility of separating fetal cfDNA from maternal plasma cfDNA based upon the size profile, but the length threshold for separation is not ~300bp, but shorter. At present, implementation of MPS makes the analysis of cfDNA size profile more unequivocal; the fetal cfDNA was reported to have a peak at approximately 143 bp with a strong 10 bp periodicity, while the dominant peak of maternal cfDNA was at approximately 166 bp and the 10 bp periodicity was extremely weak 14, 15, which was a convincing view of the size of fetal and maternal cfDNA at present. It is of note that long cfDNA fragments exist in healthy individuals, whereas next genome sequencing can only detect cfDNA fragments of <1000 bp size, and qPCR also cannot detect lengthy cfDNA fragments 5. Therefore, analyzing the entire size profile of cfDNA either by MPS or qPCR alone is insufficient in healthy individuals, especially pregnant women. A recent study found that the entire maternal plasma cfDNA fragments were ranged from 76 to 5776 bp and approximately 0.06%-0.3% of the cfDNA fragments were longer than 1000 bp by using nanopore sequencing, which could not be detected by MPS or qPCR 16. Because the application of cfDNA mainly relies on fragments of <1000 bp size, MPS is still an effective approach to accurately evaluate the size profile of cfDNA fragments despite the unavoidable limitation.

**Figure 2:**
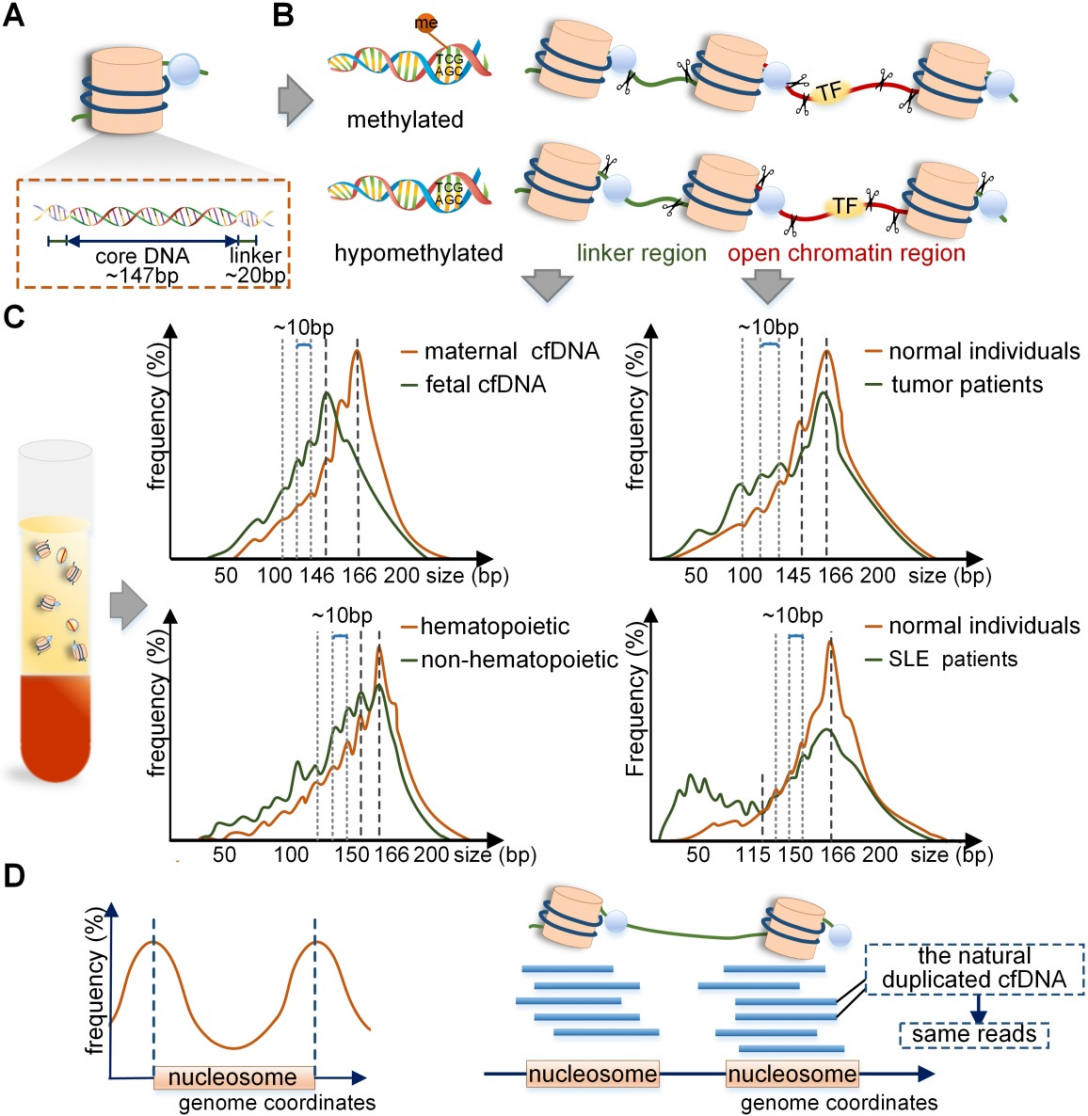
** Schematic overview of the production mechanism and size profile of cfDNA. (A)** Detailed structure of the nucleosome. **(B)** Generation process of long and short cfDNA fragments proposed in current studies. Long cfDNA fragments are cleaved at the boundary or within the linker region, but short cfDNA fragments are cut at the border of nucleosome core; hence the difference is the trimming of a 20 bp linker DNA. In open chromatin regions, DNA bound with transcription factors (TF) may be cleaved to form short fragments. **(C)** Specific size profiles of cfDNA in different populations by using MPS. The dominant peaks of fetal and maternal cfDNA fragments were at 143 bp and 166 bp, respectively. Adapted with permission from 15, copyright 2018 PNAS. The plasma cfDNA in tumor patients, transplant recipients, and SLE patients had a peak of 166 bp; short fragments were observed to be more abundant in SLE patients. Adapted with permission from 3, 22, 44, copyrights 2018 PNAS, 2014 PNAS and 2012 the Oxford University Press.** (D)** Preferred genome coordinates of cfDNA fragments are abundant at the margins of the nucleosome. Adapted with permission from 19, copyright 2019 Cold Spring Harbor Laboratory Press. The production process of naturally duplicated cfDNA* in vivo* and same reads in the sequencing phase. TF: transcription factor.

The mechanism of difference between maternal and fetal cfDNA have been analyzed in recent studies. Of note, the distribution of nucleosome DNA should be mentioned first. The nucleosome consists of a core, linker histones, and linker DNA. The nucleosome core is composed of an octamer of four types of core histone proteins winded by 147 bp of the linker DNA with a mean size of 20 bp and ranges between 0-80 bp 15 (Figure 2A). It has been reported that the fetal cfDNA is mainly cleaved at the border or within the nucleosome core, but the maternal cfDNA is mostly cut within the linker region 4; hence the difference is in the trimming of a 20 bp linker DNA (Figure 2B). Also, the 10 bp repeated periodicity was reported to be related to the structural periodicity in the helical repeat of DNA double helix 15. The main cause of different cleavage sites in maternal and fetal genomes lies in different nucleosome structures regulated by DNA methylation and histones 17. The fetal cfDNA has been shown to originate only from the hypomethylated placental tissues, while maternal cfDNA is derived from the methylated hematopoietic and hepatic tissues 18-20. The hypomethylated DNA tends to be less densely linked with histones and is more available to enzymatic degradation 21-23; therefore, the nucleosome cores in placental cells are more easily cleaved by enzymes, leading to fetal cfDNA being shorter than maternal cfDNA. The cleaving enzymes *in vivo* are complex; the caspase3- dependent enzyme and the Dnase1/3 may play an important role 24, 25. It was reported that the deletion of Dnase1/3 resulted in an increase in cfDNA fragments <120 bp and multi-nucleosomal cfDNA molecules; however, the changes only involved a small fraction of DNA molecules 25. A recent study found different effects of enzymes in the generation of cfDNA, including caspase3-dependent enzyme, Dnase1/3, and Dnase1 26, which revealed the dynamic generation mechanism of cfDNA through *in vitro* experiments for the first time.

### Tumor patients

The cfDNA in tumor patients is a mixture of cfDNA derived from tumor and non-tumor tissues, and it is a challenging endeavor to distinguish them. Although challenging, two main methods have been developed: (1) The xenografted mice model can be used to differentiate between the human tumor and non-tumor cfDNA fragments 27. (2) Since the copy number variations (CNVs) involving a whole or a large part of a chromosome arm are relatively common in tumors, for chromosome arm with amplification, the contribution of tumor cfDNA to plasma would increase, whereas, for chromosome arm with deletion, its contribution would decrease. Thus, the chromosome arm-level z-score analysis (CAZA) approach exploited by Lo *et al*. could infer tumor cfDNA based upon the information on CNVs in specific tumors 5, 28. Recently, the size profile of cfDNA in tumors of patients has been depicted more precisely by using advanced approaches (Table 1). By using AFM, 80% of cfDNA in colorectal cancer patients was found to be <145 bp 29. Mouliere *et al.* showed that the cfDNA with KRAS mutation was more fragmented than the wild-type cfDNA by qPCR in colorectal cancer patients 6. Also, by using MPS, it was reported that short cfDNA fragments preferentially carried the tumor-associated aberrations in hepatocellular carcinoma patients 28. Therefore, tumor cfDNA was considered to be shorter than non- tumor cfDNA. Precisely analyzed by MPS, the size profile of cfDNA in tumor patient was mainly peaked at 166-168bp with smaller peaks at the periodicity of 10 bp in the range of 40-166 bp 27, 28, 30 (Figure 2C).

The size of the cfDNA varies subtly with the methods, types and stages of tumors, and positions of cfDNA. With respect to various methods, using a cursory agarose gel electrophoresis, cfDNA size in tumor patients was determined to have “ladder” patterns with the main length of approximately 180 bp 31. MPS and qPCR were considered relatively robust approaches to analyze size profiles; however, using these methods, the precise size of the main peak was subtly different. The tumor cfDNA was mainly <150 bp using qPCR in xenografted mice; for instance, human cfDNA from hepatocellular carcinoma and glioblastoma was mainly at 134-144 bp and the background of mice cfDNA was distributed at 167 bp 7, 27. However, compared with qPCR, cfDNA in tumor patients measured by MPS was found to be longer and had a prominent peak at 166 bp 28. Another compelling study performed whole-genome sequencing (WGS) of cfDNA in plasma, indicating that the median overall size of cfDNA in tumor patients was around 163.8 bp 32. The reason for this variation between the two methods is unclear. A crucial difference could be that the molecules that are short or degraded with nicks in either strand can be effectively recovered through the qPCR procedures, but not readily detectable by double-stranded DNA library construction of sequencing 30, 33. The size of tumor cfDNA in plasma varies in different types and stages of tumors. It was reported that the size of cfDNA in patients with colorectal, ovarian, breast, head and neck, and melanoma cancers was shorter than that in patients with renal, glioblastoma, and bladder cancers because of the higher concentration of tumor cfDNA 34, 35. Furthermore, the cfDNA was observed to be around 176.5 bp in locally advanced pancreatic cancer patients, which was longer than 167 bp found in metastatic patients 8. Similar observations were made in breast cancer as the short cfDNA was more frequent in the plasma of metastatic than early-stage cancer patients 36. With regard to cfDNA position, analysis of cfDNA from sources other than plasma would be valuable in specific tumors, such as cerebrospinal fluid (CSF) from brain cancer patients and pleural effusion from lung cancer patients 37, 38. In a recent study, the cfDNA of a size <150 bp was reported in more than 50% of CSF samples in glioma patients, but in less than 20% of plasma samples, and the size of cfDNA in urine was the shortest compared with CSF and plasma 39. Besides, seminal cfDNA fragments longer than 1000 bp were reported to be more common in prostate cancer patients than in healthy controls 40. These results indicated different subnucleosomal fragmentation patterns of cfDNA by other nucleases in the CSF, urine, and seminal fluid.

Genome-wide hypomethylation is frequently observed in tumors, such as breast, hepatocellular carcinoma, nasopharyngeal, neuroendocrine, and lung tumors 41-43. Thus, the hypomethylation of tumor tissues may lead to higher accessibility to the enzymatic degradation and the shorter tumor cfDNA fragments, which is similar to the production mechanism of fetal cfDNA in pregnant women.

### Transplantation and systemic lupus erythematosus (SLE) patients

Recent reports have indicated that cfDNA in plasma derived from the non-hematopoietic system was shorter than that from the hematopoietic system, and a 10 bp periodicity in size below approximately 143 bp could be observed in both cases 44 (Figure 2C). Therefore, in the non-hematopoietic tissue transplant recipients, such as the liver, the donor-derived cfDNA that was cleaved mainly from the donated organs was <150 bp, and shorter than the recipient-derived cfDNA, which was from the hematopoietic system 44, 45. A recent study of sex-mismatched liver transplant recipients reported that donor-derived cfDNA was shorter than recipient-derived cfDNA quantified by using Y chromosome capture methodology 46. The situation was opposite in hematopoietic transplant recipients because the recipient-derived cfDNA originated mainly from the non-hematopoietic tissues while the donor-derived cfDNA was from the hematopoietic system. The size profile of cfDNA in SLE patients is also important. In the case of cfDNA in active SLE patients, the height of 166 bp peak reduced and the short fragments were elevated, especially for molecules <115 bp, which could contribute to more than 84% of the total cfDNA in plasma 22 (Figure 2C). It is of note that the genome-wide methylation densities in SLE groups showed significant reductions compared with those in the healthy individuals (70.1% vs. 74.3%, p<0.05) 22; thus, the production mechanism of short cfDNA in SLE was similar to that in pregnant and tumor populations.

## Application of size profile of cfDNA

### Quality control in the pre-analytical phase

**Figure 3:**
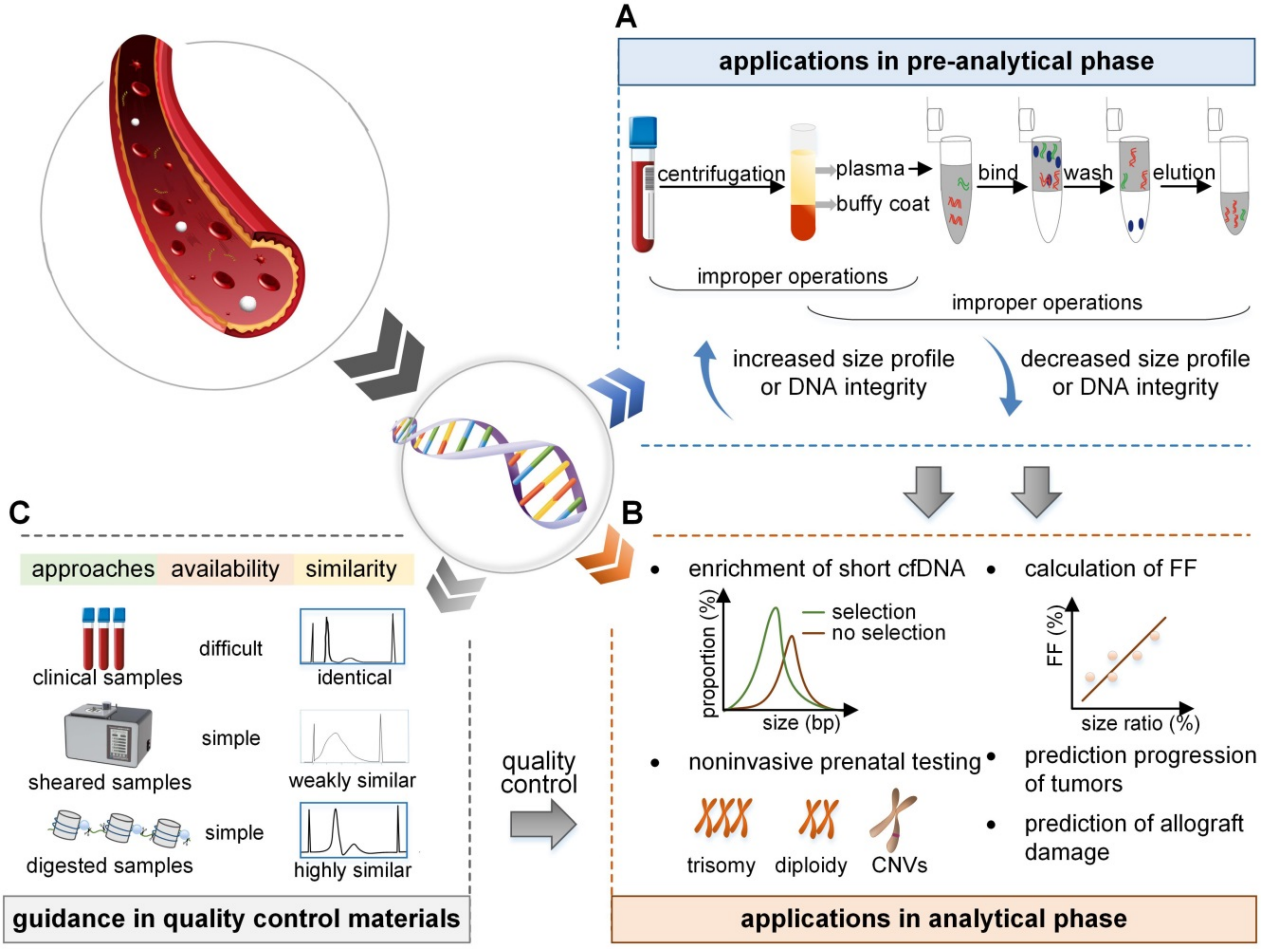
** Applications and prospects of size profile. (A)** In the pre-analytical phase, the size profile is a crucial indicator to evaluate the eligibility of cfDNA. Improper isolation of plasma leads to contamination with gDNA, and an increased size profile or DNA integrity. Improper preservation of plasma and extraction process of cfDNA lead to DNA degradation and a decreased size profile or DNA integrity.** (B)** In the analytical phase, the size profile can be applied to enrich short fragments to increase the proportion of short cfDNA. As fetal cfDNA is shorter than maternal cfDNA, the FF is linearly dependent on the size ratio; FF can be deduced by calculating the size ratio of the number of short to long cfDNA fragments. Also, the size profile of cfDNA has the potential to detect fetal abnormalities, predict tumor progression, and predict allograft damage. **(C)** Size profile is vital to assess the reliability and similarity of the quality control materials. Samples collected in the clinic, random DNA fragments induced by physical shear, and specific DNA fragments cleaved by enzymes are identical, weakly similar, and highly similar, respectively, to the real samples in size profile. FF: fetal fraction.

The size profile of cfDNA has a specific pattern in each population, and deviations from this pattern, such as increased and decreased size profile, symbolize unqualified conditions in the pre-analytical phase. The DNA integrity based on qPCR with amplicons of <80 bp and >250 bp is the recommended method for quality control in the pre-analytical phase 47 and ranges from 0.3 to 0.8 in healthy individuals. The increased DNA integrity might indicate the contamination of buffy coat, and the decreased DNA integrity (<0.1 assessed by qPCR with amplicons of 300 bp/60 bp in healthy individuals) implies degraded samples 29, 48 (Figure 3).

#### Increased size profile of cfDNA

The proper size profile of cfDNA after extraction should have a dominant peak of around 146-166 bp with no contamination of genomic DNA (gDNA); however, the increase in size profile is mainly due to leukocyte lysis, leading to the release of gDNA and an increase in long fragments. Improper selection of specimen types and separation of plasma are the main reasons. First, plasma is an ideal sample type for cfDNA detection compared to serum as clotting during the extraction of serum significantly increases the observed size of cfDNA 48. Besides, the improper selection of collection tubes can lead to contamination of gDNA 49. Last, isolation operations of plasma should also be of concern because a delayed separation of plasma and a low-speed protocol coincide with the contamination of gDNA 50. Several studies have been employed to avoid this phenomenon, for the selection of specimen types, plasma should be the best type 48, and cell stabilizing tubes should be used whenever possible; for instance, cfDNA was reported to be more stable in Streck tubes than in the BD Vacutainer K_2_EDTA tubes 51. More importantly, blood tubes should be placed upright to reduce hemolysis, sloshing should be avoided, and temperature fluctuations should be minimized 52. During the isolation phase, blood samples should be stored at 4℃ and be processed within 4 h after collection 53. If the isolation needs to be delayed, the samples should be stored at 4℃ in a K_2_EDTA tube for one day 54. Besides, a two-step and high-speed plasma separation procedure is required, it is precisely recommended to be 1600 × g for 10 min at 4°C and 16000 × g for 10 min at 4°C 47. Also, the samples should be removed carefully after the first centrifugation to avoid contamination by the buffy coat during the separation of plasma 47.

#### Decreased size profile of cfDNA

A decrease in the size profile is mainly due to the degradation of cfDNA in the samples. After the collection of blood specimens, the repeated freeze-thaw cycles of plasma reduce the DNA integrity 48. Also, nuclease contamination is an indispensable factor that influences the size profile of cfDNA 47. During the cfDNA extraction procedure, the ability of various methods to bind small fragments of cfDNA varies, resulting in a bias in the extracted DNA fragments. The extraction methods generally consist of columns, magnetic particles, and precipitation-based methods. It has been reported that precipitation-based methods generally generate relatively less DNA fragmentation 55. To standardize the preservation and extraction process, Meddeb *et al.* suggested that the plasma should be preserved for the final experimental purpose without repeated freeze-thaw cycles 47. Appropriate selection of an extraction kit is essential 56 and the cfDNA extracts should be stored at -20°C or -80°C to reduce degradation 47. If the DNA integrity is calculated to be less than 0.1 in healthy individuals, cfDNA is considered to be degraded in samples 34 and cannot be used for subsequent experiments.

### Assay innovations in the analytical phase

#### Size-based enrichment for cfDNA

It is noteworthy that low levels of fetal or tumor cfDNA always result in detection failure in NIPT or liquid biopsy, so the enrichment of short fragments is vital in the workflow. Generally, selection of short fragments can be conducted before and after the sequencing phase. Before sequencing, the PCR analysis based on short and long amplicons is an efficient way. As short amplicons can bind long and short cfDNA fragments, whereas long amplicons only can bind long fragments, therefore, using shorter amplicons will bind more interested fragments. Compared to conventional assays, the amount of fetal cfDNA was almost 1.6 times higher when short amplicons of 50 bp were used 57. In another report, when PCR was performed based on short amplicons of 64 bp, the amount of FF was successfully increased from 18% to 38% 43. In addition, single-stranded library preparation and hybrid-capture (SLHC) is also a robust selection approach before sequencing. Some of cfDNA fragments in tumor patients are degraded with nicks in the strand, which cannot be captured by the conventional method. The SLHC first denatures cfDNA into single-stranded fragments after end repair, then library construction of the single-stranded fragments is performed, including the short degraded cfDNA 33, 58. It has been shown that SLHC could efficiently recover and enrich the short cfDNA fragments (<100 bp) to attain a higher detection rate from 45% to 75% 33. After sequencing, the *in silico* size selection was applied during the read-pair positioning process. Once these unprocessed sequencing reads were mapped to the reference, the method selected the interesting reads ranging from 90 bp to 150 bp, leading to a 2-fold enrichment in 95% of tumor patients 35. The selection of short cfDNA increases the relative abundance of fetal or tumor fragments, despite the potential loss of long fragments, but this limitation has not been fully discussed in the literature. In summary, the size profile plays an indispensable role in the enrichment, thereby increasing the level of cfDNA of interest and decreasing the rate of detection failure.

#### Same cleavage pattern

For the exploration of specific end coordinates of cfDNA fragments, the windowed protection score (WPS) is an available assessment approach. WPS refers to the number of cfDNA fragments that have no endpoints in a given 120 bp genome window minus those with endpoints in that window 18. By WPS analysis, cfDNA fragments have a stable cleavage pattern with the endpoints intensively clustering adjacent to the boundary of the nucleosome core or the linker region 3, 18. Therefore, cfDNA fragments have series of preferred genome coordinates, but these sites on the sides of nucleosome DNA are limited, thus producing a few fragments of the same length and breakpoint. That is, cfDNA in plasma has the characteristic of natural duplication to a certain degree (Figure 2D). Moreover, the artificially duplicated cfDNA fragments are introduced by PCR during the library construction 59, and the background errors of bases are generated, which is a serious barrier to the liquid biopsy. Another severe barrier is the low level of mutant tumor cfDNA in patients 60. In the sequencing phase, to reduce errors, the repeat cfDNA is removed, including the naturally occurring repeat cfDNA fragments as they are not specially tagged, exacerbating the low concentration of cfDNA, leading to a level in many patients much lower than the detection threshold.

Based on the cleavage pattern and characteristics of cfDNA, Newman *et al.* recognized the importance of identifying naturally duplicated cfDNA. They designed the molecular barcode technology to add a unique molecular index (UMI) at the ends of the cfDNA fragments, where the UMI is sufficiently diverse to ensure that each cfDNA molecule can be labeled differently 59. Naturally repeated DNA fragments are not removed when filtering the errors because of the presence of barcodes, leading to a 15-fold reduced error rate and an improvement of error-free regions from 90% to 98% 59. Consequently, cfDNA fragments with the same size and endpoints indicate naturally repeated fragments, guiding the utilization of unique barcodes during de-duplication.

#### Calculation of FF

The FF calculation is an indispensable part of NIPT, and several calculation methods based on chromosomes Y and X, SNPs, and seq FF have been developed recently 61; the size-based and nucleosome track-based approach can also be used to calculate FF. As fetal cfDNA is shorter than maternal cfDNA, the size-based method mainly calculates the relative proportions of short and long (100-150 bp and 163-169 bp, respectively) cfDNA fragments to determine the FF 13. The FF thus derived is highly consistent with the ratio determined by the Y chromosome sequence (r=0.827, p<0.0001). In summary, the size-based method performs shallow depth sequencing of maternal plasma DNA and is moderately accurate in conventional NIPT.

Another method of cfDNA size profile is the nucleosome track-based approach, which is based on the different start sites of sequence reads from fetal and maternal cfDNA fragments because only maternal cfDNA involves linker DNA 62. In the sequencing data, the reads involving linker DNA could be clearly recognized by identifying those that start in the regions over 73 bp upstream and downstream of the nucleosome core 62. Therefore, the frequency of reads involving linker DNA can be exploited to calculate the FF 14. However, compared with the size-based method, the correlation between this method and the chromosome Y-based method is low (r=0.636). This method is cost-effective and does not rely on fetal gender, but the accuracy should be further developed in the future 61.

#### Application in the detection of aneuploidy

Traditional noninvasive prenatal detection of aneuploidy mainly includes count-based methods of chromosomes and single nucleotide polymorphism (SNP)-based methods 63; the size-based method is also a potential and novel approach. In theory, when extra copies of fetal chromosomes are present, the relative proportion of cfDNA produced by that chromosome increases shortening the size profile of this chromosomal cfDNA in plasma. Therefore, the size-based method calculates the ratio of cfDNA fragments with short sizes (e.g., <150 bp) to all the sequenced fragments from the targeted chromosome in the sample, followed by comparison with the reference proportion in diploid pregnant women to acquire the z-score 13. If the size of cfDNA fragments of a chromosome in the sample is significantly shorter than the expected value (e.g., z-score >3), the risk of trisomy of this chromosome is higher 64. As the count analysis of chromosomes is the common method for detecting aneuploidy, combining count- and size-based z-score should be an accurate and rigorous scheme. Zhang *et al.* combined count-based method with size-based algorithms to obtain a more accurate z-score to facilitate the detection for fetal trisomy. When 180 cases were tested by this combination method, the sensitivity and specificity increased from 75% to 80% and 98.86% to 99.43% after the size-based correction of 100 bp 65. Besides, the sensitivity and specificity of count-based z-score with 130 bp size-based corrections reached up to 100%, which were more efficient than the correction of 100 bp. Therefore, the combination of count- and size-based analysis would enhance the detection of fetal aneuploidy in NIPT 65, 66. However, determining the specific size value of the analysis is urgently needed. Also, the current method can detect fetal aneuploidy with a cut-off value of 3-4% FF 61; therefore, lower FF plasma samples should be considered when confirming the performance of the size-based method in the future.

#### Application in the detection of CNVs

The principle of the size-based method of fetal aneuploidies can also be used in the detection of CNVs. Since the size of fetal cfDNA is shorter than that of the mother, the presence of fetal micro-deletion or micro-duplication would lengthen or shorten the size profile of cfDNA released from that chromosome in the maternal plasma 61. As was the case with aneuploidy detection, after WGS or targeted sequencing, the ratio of cfDNA fragments with short sizes (e.g., <150 bp) of the target chromosome in the sample was calculated and compared with the ratio of reference to acquire the z-score 67. The result indicated that the size-based algorithm correctly identified 17 out of 18 cases with CNVs ranging between 3-40 Mb 67. The sensitivity and specificity have not been studied in large-scale experiments. The size-based method is feasible in theory but has not been widely verified in many studies. Therefore, at present, the combination of traditional and size-based methods is an effective and comprehensive approach to detect fetal CNVs.

#### Potential application in liquid biopsy

Tumor cfDNA with the mutation information was reported to have the potential to detect tumors and predict drug therapy 68, 69. Similarly, the size of cfDNA in tumor patients has been shown to be shorter in advanced tumor stages and metastasis, and can be applied to monitor the evolutionary dynamics and prognosis of tumors. It has been reported that the cfDNA size in healthy control samples was longer (mean 176.5 bp, range 168-185 bp) than that in local pancreatic cancer samples (mean 170 bp, range 167-173 bp, p=0.001), and was the shortest in metastatic patients (mean 167 bp, range 148-180 bp, p<0.001) 36, indicating that the size of cfDNA was highly related to the progression and metastasis of tumors. The data also indicated that a fragment size of <167 bp before treatment was significantly associated with shorter progression-free survival (p=0.002) and overall survival (p=0.001) 36. Similarly, the ratio of short (50-166 bp) to large (167-250 bp) cfDNA fragments had a significant association with poor survival in renal cell carcinoma 70 as well as in hepatocellular carcinoma, prostate, and primary and metastatic breast cancer patients 28, 37, 71. The analysis of cfDNA size profile in the early stages of the tumor by calculating the ratio of short to long fragments or calculating DNA integrity is a simple, rapid, and economical method to determine prognostic information. Analysis of size profile to predict the progression of the tumor has only been verified in some tumors, and it is worthwhile to evaluate other tumors. In conclusion, cfDNA size profile may be a potential biomarker for monitoring the prognosis of tumors.

#### Potential application in transplantation and other diseases

Accurate and early assessment of allograft damage is vital for the long-term survival of transplant patients. Recently, several prospective studies have proven the high amount of cfDNA derived from grafts was associated with the allograft rejection in liver and kidney transplantation 72, 73. However, little information is available on the relationship between cfDNA size and allograft damage. The cfDNA derived from the graft is shorter than that from hematopoietic cells, so the increased proportion of short cfDNA is speculated to herald allograft rejection. It was reported that a high ratio of short (105-145 bp) to long cfDNA fragments (160-170 bp) in liver transplantation points towards an early trend of allograft damage. Also, the assessment of allograft damage based on the ratio of short to long cfDNA fragments was highly consistent with that based on the cfDNA quantified by chromosome Y (p<0.0001), and routine liver function enzymes (p<0.0001) 46. Therefore, the size analysis of cfDNA derived from the graft may be a potential approach to assess allograft damage, for which large-scale analysis and validation are needed. Besides, the quantification of cfDNA has been shown to be related to autoimmune diseases and myocardial infarction 74, 75. Additional studies about the relationship between the size profile of cfDNA and these diseases may be valuable.

### Guidance in preparation of QCMs

Although recent advances have improved the performance of NIPT and liquid biopsy, it is still challenging because of the low concentrations of cfDNA, varied detection settings, and complex workflows. Thus, there is an urgent requirement for QCMs for proficiency tests and quality control 76, 77. Currently, several QCMs are available for cfDNA analysis, such as clinical samples for the College of American Pathologists in their performance testing programs, ultrasonically interrupted samples 78, and samples digested by enzymes 79. The size profile of cfDNA is an important indicator to evaluate whether the QCMs successfully simulate the real samples (Figure 3).

Samples from the clinic reflect the characteristics of real cfDNA, but the acquisition is poor. Random DNA fragmentation induced by physical shear results in a broad size profile and a random distribution pattern, which is different from the real cfDNA and does not reflect its accurate signature. However, with MNase digestion technology, the QCMs for tumor cfDNA had a dominant peak of 147 bp, which successfully simulated real tumor cfDNA *in vitro* 79. Furthermore, based on the differences in maternal and fetal cfDNA size and cleavage sites, the QCMs for NIPT digested by DNA fragmentation factor (DFF) and MNase contained a mixture of DNA with the dominant peak of 162 and 146 bp, respectively, which successfully simulated the maternal and fetal cfDNA fragments 24, 80. Therefore, the size profile is a vital indicator to assess and evaluate the eligibility of QCMs. With the exploration of the crucial mechanism of cfDNA, the materials will be prepared carefully to better resemble the real samples.

## Conclusions

The past few decades have witnessed rapid improvement in the comprehension of cfDNA size profile and its application in precision medicine 79. The size profile of cfDNA is applied widely in NIPT, liquid biopsy, and QCMs in the laboratory. In the pre-analytical phase, the size profile serves as a vital indicator to evaluate the eligibility of specimens and to ensure the successful implementation of experiments. In the analytical phase, size profile can be applied for the enrichment of short fragments, calculation of FF, detection of fetal abnormalities, prediction of progress in tumors and graft rejection in transplantation. It also plays an important role in evaluating the similarity between QCMs and real samples. Thus, the advantages of cfDNA have inspired and broadened its applications in a variety of areas.

Studies on the size profile of cfDNA have paved way for understanding the mechanism of its generation. The tissue-of-origin and footprint analysis of cfDNA are also the hotspots of current research and are expected to expand the current understanding and facilitate its implementation for novel assays 19. Some of the applications of size profile are only at an exploratory stage. For instance, cfDNA size profile might be applied to predict the progression and prognosis of tumors and serve as a novel diagnostic indicator for transplantation, myocardial infarction, SLE, and severe sepsis. However, these clinical applications await long-term validation studies. Despite the obstacles and the unknowns, the size profile of cfDNA fragments still has a good prospect to guide innovative new assays and provide hope for precision medicine.

## Figures and Tables

**Table 1:** Size profile of cfDNA fragments in different populations

People		Sample	Size profile	Technology	Reference
Pregnant women	Not mentioned	Blood	<313 bp (fetal cfDNA) and 145-201 bp (plasma cfDNA)	qPCR (SRY, LEP)	9
	12 weeks of gestation		143 bp (fetal cfDNA) and 166 bp (maternal cfDNA)	MPS	14
	Median 13 weeks of gestation		<150 bp (fetal cfDNA)	MPS and microchip-based capillary electrophoresis	13
	Median 33 or 32 weeks of gestation		≤107 bp (60% of fetal cfDNA)	qPCR (SRY)	82
	Carry fetus of aneuploidy		169 bp, 176 bp (plasma cfDNA)	MPS	64
	Head and neck cancer patients		400 bp/100 bp amplicons	qPCR (ACTB)	83
	Nasopharyngeal carcinoma patients		201 bp/105 bp amplicons	qPCR (LEP)	84
	Colorectal cancer patients		<145 bp	AFM	29
	Hepatocellular cancer patients		166 bp	MPS	28
	Animal models(hepatocellular cancer and glioblastoma multiforme)		134 bp (tumor cfDNA) and 167 bp (rat cfDNA)	Xenotransplantation and MPS	27
	Animal models(melanoma)		145 bp (tumor cfDNA) and 165 bp (rat cfDNA)	Xenotransplantation and MPS	27
	Animal models (colon cancer)		<150 bp (tumor cfDNA)	Xenotransplantation and qPCR (KRAS)	7
	Prostate cancer patients	Seminal fluid	Smears ranged from 250 bp to 10,000 bp	Agarose gel electrophoresis	85
	Stage III and IV lung cancer patients		166 bp	MPS	30
Transplantation	Six hematopoietic stem cell transplant and one liver transplant recipients	Blood	≤150 bp (non-hematopoietically derived cfDNA)	MPS	43
Other diseases	Sepsis patients	Blood	Around 150 bp and 300 bp	Agarose gel electrophoresis	86
	SLE patients		<115 bp (84% of total cfDNA)	MPS	22

**Notes:** AFM: atomic force microscopy; MPS: massively parallel sequencing; qPCR: quantitative real-time PCR; SLE: slystemic lupus erythematosus.

## Abbreviations

AFM: atomic force microscopy
cfDNA: cell-free DNA
CNVs: copy number variations
CAZA: chromosome arm-level z-score analysis
CSF: cerebrospinal fluid
DFF: DNA fragmentation factor
FF: fetal fraction
gDNA: genomic DNA
MPS: massively parallel sequencing
MNase: micrococcal nuclease
NIPT: noninvasive prenatal testing
qPCR: quantitative real-time PCR
QCMs: quality control materials
SLE: systemic lupus erythematosus
SLHC: single-strand library preparation and hybrid-capture
SNP: single nucleotide polymorphism
TF: transcriptional factor
UMI: unique molecular index
WGS: whole genome sequencing
WPS: windowed protection score

## References

[1] Mandel P, Metais P (1948). Les acides nucléiques du plasma sanguin chez l'homme. C R Seances Soc Biol Fil.

[2] Lo YM, Corbetta N, Chamberlain PF, Rai V, Sargent IL, Redman CW (1997). Presence of fetal DNA in maternal plasma and serum. Lancet.

[3] Jiang P, Sun K, Tong YK, Cheng SH, Cheng THT, Heung MMS (2018). Preferred end coordinates and somatic variants as signatures of circulating tumor DNA associated with hepatocellular carcinoma. Proc Natl Acad Sci U S A.

[4] Chan KC, Jiang P, Sun K, Cheng YK, Tong YK, Cheng SH (2016). Second generation noninvasive fetal genome analysis reveals de novo mutations, single-base parental inheritance, and preferred DNA ends. Proc Natl Acad Sci U S A.

[5] Jiang P, Lo YMD (2016). The long and short of circulating cell-free DNA and the ins and outs of molecular diagnostics. Trends Genet.

[6] Mouliere F, El Messaoudi S, Gongora C, Guedj AS, Robert B, Del Rio M (2013). Circulating cell-free DNA from colorectal cancer patients may reveal high kras or braf mutation load. Transl Oncol.

[7] Mouliere F, Robert B, Arnau Peyrotte E, Del Rio M, Ychou M, Molina F (2011). High fragmentation characterizes tumour-derived circulating DNA. PloS one.

[8] Lapin M, Oltedal S, Tjensvoll K, Buhl T, Smaaland R, Garresori H (2018). Fragment size and level of cell-free DNA provide prognostic information in patients with advanced pancreatic cancer. J Transl Med.

[9] Chan KC, Zhang J, Hui AB, Wong N, Lau TK, Leung TN (2004). Size distributions of maternal and fetal DNA in maternal plasma. Clin Chem.

[10] Chim SS, Tong YK, Chiu RW, Lau TK, Leung TN, Chan LY (2005). Detection of the placental epigenetic signature of the maspin gene in maternal plasma. Proc Natl Acad Sci U S A.

[11] Li Y, Zimmermann B, Rusterholz C, Kang A, Holzgreve W, Hahn S (2004). Size separation of circulatory DNA in maternal plasma permits ready detection of fetal DNA polymorphisms. Clin Chem.

[12] Fan HC, Blumenfeld YJ, Chitkara U, Hudgins L, Quake SR (2010). Analysis of the size distributions of fetal and maternal cell-free DNA by paired-end sequencing. Clinical chemistry.

[13] Yu SC, Chan KC, Zheng YW, Jiang P, Liao GJ, Sun H (2014). Size-based molecular diagnostics using plasma DNA for noninvasive prenatal testing. Proc Natl Acad Sci U S A.

[14] Lo YM, Chan KC, Sun H, Chen EZ, Jiang P, Lun FM (2010). Maternal plasma DNA sequencing reveals the genome-wide genetic and mutational profile of the fetus. Science translational medicine.

[15] Sun K, Jiang P, Wong AIC, Cheng YKY, Cheng SH, Zhang H (2018). Size-tagged preferred ends in maternal plasma DNA shed light on the production mechanism and show utility in noninvasive prenatal testing. Proc Natl Acad Sci U S A.

[16] Cheng SH, Jiang P, Sun K, Cheng YK, Chan KC, Leung TY (2015). Noninvasive prenatal testing by nanopore sequencing of maternal plasma DNA: Feasibility assessment. Clin Chem.

[17] Lun FM, Chiu RW, Sun K, Leung TY, Jiang P, Chan KC (2013). Noninvasive prenatal methylomic analysis by genomewide bisulfite sequencing of maternal plasma DNA. Clin Chem.

[18] Snyder MW, Kircher M, Hill AJ, Daza RM, Shendure J (2016). Cell-free DNA comprises an in vivo nucleosome footprint that informs its tissues-of-origin. Cell.

[19] Sun K, Jiang P, Cheng SH, Cheng THT, Wong J, Wong VWS (2019). Orientation-aware plasma cell-free DNA fragmentation analysis in open chromatin regions informs tissue of origin. Genome Res.

[20] Sun K, Jiang P, Chan KC, Wong J, Cheng YK, Liang RH (2015). Plasma DNA tissue mapping by genome-wide methylation sequencing for noninvasive prenatal, cancer, and transplantation assessments. Proc Natl Acad Sci U S A.

[21] Kelly TK, Liu Y, Lay FD, Liang G, Berman BP, Jones PA (2012). Genome-wide mapping of nucleosome positioning and DNA methylation within individual DNA molecules. Genome Res.

[22] Chan RW, Jiang P, Peng X, Tam LS, Liao GJ, Li EK (2014). Plasma DNA aberrations in systemic lupus erythematosus revealed by genomic and methylomic sequencing. Proc Natl Acad Sci U S A.

[23] Xiang Y, Zhang J, Li Q, Zhou X, Wang T, Xu M (2014). DNA methylome profiling of maternal peripheral blood and placentas reveal potential fetal DNA markers for non-invasive prenatal testing. Mol Hum Reprod.

[24] Zhang R, Ding J, Gao P, Li Z, Tan P, Li J (2019). Generation of highly biomimetic quality control materials for noninvasive prenatal testing based on enzymatic digestion of matched mother-child cell lines. Clin Chem.

[25] Serpas L, Chan RWY, Jiang P, Ni M, Sun K, Rashidfarrokhi A (2019). Dnase1/3 deletion causes aberrations in length and end-motif frequencies in plasma DNA. Proc Natl Acad Sci U S A.

[26] Han DSC, Ni M, Chan RWY, Chan VWH, Lui KO, Chiu RWK (2020). The Biology of Cell-free DNA Fragmentation and the Roles of DNASE1, DNASE1L3, and DFFB. Am J Hum Genet.

[27] Underhill HR, Kitzman JO, Hellwig S, Welker NC, Daza R, Baker DN (2016). Fragment length of circulating tumor DNA. PLoS Genet.

[28] Jiang P, Chan CW, Chan KC, Cheng SH, Wong J, Wong VW (2015). Lengthening and shortening of plasma DNA in hepatocellular carcinoma patients. Proc Natl Acad Sci U S A.

[29] Mouliere F, El Messaoudi S, Pang D, Dritschilo A, Thierry AR (2014). Multi-marker analysis of circulating cell-free DNA toward personalized medicine for colorectal cancer. Mol Oncol.

[30] Sanchez C, Snyder MW, Tanos R, Shendure J, Thierry AR (2018). New insights into structural features and optimal detection of circulating tumor DNA determined by single-strand DNA analysis. NPJ Genom Med.

[31] Jahr S, Hentze H, Englisch S, Hardt D, Fackelmayer FO, Hesch RD (2001). DNA fragments in the blood plasma of cancer patients: Quantitations and evidence for their origin from apoptotic and necrotic cells. Cancer Res.

[32] Cristiano S, Leal A, Phallen J, Fiksel J, Adleff V, Bruhm DC (2019). Genome-wide cell-free DNA fragmentation in patients with cancer. Nature.

[33] Liu X, Liu L, Ji Y, Li C, Wei T, Yang X (2019). Enrichment of short mutant cell-free DNA fragments enhanced detection of pancreatic cancer. EBioMedicine.

[34] Bettegowda C, Sausen M, Leary RJ, Kinde I, Wang Y, Agrawal N (2014). Detection of circulating tumor DNA in early- and late-stage human malignancies. Sci Transl Med.

[35] Mouliere F, Chandrananda D, Piskorz AM, Moore EK, Morris J, Ahlborn LB (2018). Enhanced detection of circulating tumor DNA by fragment size analysis. Sci Transl Med.

[36] Madhavan D, Wallwiener M, Bents K, Zucknick M, Nees J, Schott S (2014). Plasma DNA integrity as a biomarker for primary and metastatic breast cancer and potential marker for early diagnosis. Breast Cancer Res Treat.

[37] von Baumgarten L, Kumbrink J, Jung A, Reischer A, Flach M, Liebmann S (2020). Therapeutic management of neuro-oncologic patients - potential relevance of CSF liquid biopsy. Theranostics.

[38] Tong L, Ding N, Tong X, Li J, Zhang Y, Wang X (2019). Tumor-derived DNA from pleural effusion supernatant as a promising alternative to tumor tissue in genomic profiling of advanced lung cancer. Theranostics.

[39] Mouliere F, Mair R, Chandrananda D, Marass F, Smith CG, Su J (2018). Detection of cell-free DNA fragmentation and copy number alterations in cerebrospinal fluid from glioma patients. EMBO Mol Med.

[40] Ponti G, Maccaferri M, Manfredini M, Micali S, Torricelli F, Milandri R (2019). Quick assessment of cell-free DNA in seminal fluid and fragment size for early non-invasive prostate cancer diagnosis. Clin Chim Acta.

[41] Ushijima T (2005). Detection and interpretation of altered methylation patterns in cancer cells. Nat Rev Cancer.

[42] Chan KC, Jiang P, Chan CW, Sun K, Wong J, Hui EP (2013). Noninvasive detection of cancer-associated genome-wide hypomethylation and copy number aberrations by plasma DNA bisulfite sequencing. Proc Natl Acad Sci U S A.

[43] Hlady RA, Zhao X, Pan X, Yang JD, Ahmed F, Antwi SO (2019). Genome-wide discovery and validation of diagnostic DNA methylation-based biomarkers for hepatocellular cancer detection in circulating cell free DNA. Theranostics.

[44] Zheng YW, Chan KC, Sun H, Jiang P, Su X, Chen EZ (2012). Nonhematopoietically derived DNA is shorter than hematopoietically derived DNA in plasma: A transplantation model. Clin Chem.

[45] Lui YY, Woo KS, Wang AY, Yeung CK, Li PK, Chau E (2003). Origin of plasma cell-free DNA after solid organ transplantation. Clin Chem.

[46] Ng HI, Zhu X, Xuan L, Long Y, Mao Y, Shi Y (2019). Analysis of fragment size distribution of cell-free DNA: A potential non-invasive marker to monitor graft damage in living-related liver transplantation for inborn errors of metabolism. Mol Genet Metab.

[47] Meddeb R, Pisareva E, Thierry AR (2019). Guidelines for the preanalytical conditions for analyzing circulating cell-free DNA. Clin Chem.

[48] Chan KC, Yeung SW, Lui WB, Rainer TH, Lo YM (2005). Effects of preanalytical factors on the molecular size of cell-free DNA in blood. Clin Chem.

[49] Merker JD, Oxnard GR, Compton C, Diehn M, Hurley P, Lazar AJ (2018). Circulating tumor DNA analysis in patients with cancer: American society of clinical oncology and college of american pathologists joint review. J Clin Oncol.

[50] Sorber L, Zwaenepoel K, Jacobs J, De Winne K, Goethals S, Reclusa P (2019). Circulating cell-free DNA and rna analysis as liquid biopsy: Optimal centrifugation protocol. Cancers (Basel).

[51] Norton SE, Lechner JM, Williams T, Fernando MR (2013). A stabilizing reagent prevents cell-free DNA contamination by cellular DNA in plasma during blood sample storage and shipping as determined by digital pcr. Clin Biochem.

[52] Malentacchi F, Pizzamiglio S, Verderio P, Pazzagli M, Orlando C, Ciniselli CM (2015). Influence of storage conditions and extraction methods on the quantity and quality of circulating cell-free DNA (ccfdna): The spidia-dnaplas external quality assessment experience. Clin Chem Lab Med.

[53] El Messaoudi S, Rolet F, Mouliere F, Thierry AR (2013). Circulating cell free DNA: Preanalytical considerations. Clin Chim Acta.

[54] Jung M, Klotzek S, Lewandowski M, Fleischhacker M, Jung K (2003). Changes in concentration of DNA in serum and plasma during storage of blood samples. Clin Chem.

[55] Malentacchi F, Pizzamiglio S, Ibrahim-Gawel H, Pazzagli M, Verderio P, Ciniselli CM (2016). Second spidia-DNA external quality assessment (eqa): Influence of pre-analytical phase of blood samples on genomic DNA quality. Clin Chim Acta.

[56] Fong SL, Zhang JT, Lim CK, Eu KW, Liu Y (2009). Comparison of 7 methods for extracting cell-free DNA from serum samples of colorectal cancer patients. Clin Chem.

[57] Sikora A, Zimmermann BG, Rusterholz C, Birri D, Kolla V, Lapaire O (2010). Detection of increased amounts of cell-free fetal DNA with short pcr amplicons. Clin Chem.

[58] Vong JSL, Tsang JCH, Jiang P, Lee WS, Leung TY, Chan KCA (2017). Single-stranded DNA library preparation preferentially enriches short maternal DNA in maternal plasma. Clin Chem.

[59] Newman AM, Lovejoy AF, Klass DM, Kurtz DM, Chabon JJ, Scherer F (2016). Integrated digital error suppression for improved detection of circulating tumor DNA. Nat Biotechnol.

[60] Wan JCM, Massie C, Garcia-Corbacho J, Mouliere F, Brenton JD, Caldas C (2017). Liquid biopsies come of age: Towards implementation of circulating tumour DNA. Nat Rev Cancer.

[61] Peng XL, Jiang P (2017). Bioinformatics approaches for fetal DNA fraction estimation in noninvasive prenatal testing. Int J Mol Sci.

[62] Straver R, Oudejans CB, Sistermans EA, Reinders MJ (2016). Calculating the fetal fraction for noninvasive prenatal testing based on genome-wide nucleosome profiles. Prenat Diagn.

[63] Shi J, Zhang R, Li J, Zhang R (2019). Novel perspectives in fetal biomarker implementation for the noninvasive prenatal testing. Crit Rev Clin Lab Sci.

[64] Fan HC, Blumenfeld YJ, Chitkara U, Hudgins L, Quake SR (2008). Noninvasive diagnosis of fetal aneuploidy by shotgun sequencing DNA from maternal blood. Proc Natl Acad Sci U S A.

[65] Zhang L, Zhu Q, Wang H, Liu S (2017). Count-based size-correction analysis of maternal plasma DNA for improved noninvasive prenatal detection of fetal trisomies 13, 18, and 21. Am J Transl Res.

[66] Budis J, Gazdarica J, Radvanszky J, Szucs G, Kucharik M, Strieskova L (2019). Combining count- and length-based z-scores leads to improved predictions in non-invasive prenatal testing. Bioinformatics.

[67] Zhao C, Tynan J, Ehrich M, Hannum G, McCullough R, Saldivar JS (2015). Detection of fetal subchromosomal abnormalities by sequencing circulating cell-free DNA from maternal plasma. Clin Chem.

[68] Jiang T, Li X, Wang J, Su C, Han W, Zhao C (2017). Mutational Landscape of cfDNA Identifies Distinct Molecular Features Associated With Therapeutic Response to First-Line Platinum-Based Doublet Chemotherapy in Patients with Advanced NSCLC. Theranostics.

[69] Berger AW, Schwerdel D, Reinacher-Schick A, Uhl W, Algül H, Friess H (2019). A Blood-Based Multi Marker Assay Supports the Differential Diagnosis of Early-Stage Pancreatic Cancer. Theranostics.

[70] Yamamoto Y, Uemura M, Fujita M, Maejima K, Koh Y, Matsushita M (2019). Clinical significance of the mutational landscape and fragmentation of circulating tumor DNA in renal cell carcinoma. Cancer Sci.

[71] Hanley R, Rieger-Christ KM, Canes D, Emara NR, Shuber AP, Boynton KA (2006). DNA integrity assay: A plasma-based screening tool for the detection of prostate cancer. Clin Cancer Res.

[72] Schutz E, Fischer A, Beck J, Harden M, Koch M, Wuensch T (2017). Graft-derived cell-free DNA, a noninvasive early rejection and graft damage marker in liver transplantation: A prospective, observational, multicenter cohort study. PLoS Med.

[73] Oellerich M, Shipkova M, Asendorf T, Walson PD, Schauerte V, Mettenmeyer N (2019). Absolute quantification of donor-derived cell-free DNA as a marker of rejection and graft injury in kidney transplantation: Results from a prospective observational study. Am J Transplant.

[74] Zhong XY, von Muhlenen I, Li Y, Kang A, Gupta AK, Tyndall A (2007). Increased concentrations of antibody-bound circulatory cell-free DNA in rheumatoid arthritis. Clin Chem.

[75] Antonatos D, Patsilinakos S, Spanodimos S, Korkonikitas P, Tsigas D (2006). Cell-free DNA levels as a prognostic marker in acute myocardial infarction. Ann N Y Acad Sci.

[76] Gargis AS, Kalman L, Berry MW, Bick DP, Dimmock DP, Hambuch T (2012). Assuring the quality of next-generation sequencing in clinical laboratory practice. Nat Biotechnol.

[77] Tsang JCH, Chan KCA (2017). Quality materials for quality assurance in the analysis of liquid biopsy samples. Clin Chem.

[78] Zhang R, Zhang H, Li Y, Han Y, Xie J, Li J (2016). External quality assessment for detection of fetal trisomy 21, 18, and 13 by massively parallel sequencing in clinical laboratories. J Mol Diagn.

[79] Zhang R, Peng R, Li Z, Gao P, Jia S, Yang X (2017). Synthetic circulating cell-free DNA as quality control materials for somatic mutation detection in liquid biopsy for cancer. Clin Chem.

[80] Sistermans EA (2019). The importance of reliable quality control materials for noninvasive prenatal testing. Clin Chem.

[81] Ashley EA (2016). Towards precision medicine. Nat Rev Genet.

[82] Koide K, Sekizawa A, Iwasaki M, Matsuoka R, Honma S, Farina A (2005). Fragmentation of cell-free fetal DNA in plasma and urine of pregnant women. Prenat Diagn.

[83] Jiang WW, Zahurak M, Goldenberg D, Milman Y, Park HL, Westra WH (2006). Increased plasma DNA integrity index in head and neck cancer patients. Int J Cancer.

[84] Ponti G, Maccaferri M, Mandrioli M, Manfredini M, Micali S, Cotugno M (2018). Seminal cell-free DNA assessment as a novel prostate cancer biomarker. Pathol Oncol Res.

[85] Dwivedi DJ, Toltl LJ, Swystun LL, Pogue J, Liaw KL, Weitz JI (2012). Prognostic utility and characterization of cell-free DNA in patients with severe sepsis. Crit Care.

[86] Chan KC, Leung SF, Yeung SW, Chan AT, Lo YM (2008). Persistent aberrations in circulating DNA integrity after radiotherapy are associated with poor prognosis in nasopharyngeal carcinoma patients. Clin Cancer Res.

